# Genetic Variations in the *NRF2* Microsatellite Contribute to the Regulation of Bovine Sperm-Borne Antioxidant Capacity

**DOI:** 10.3390/cells13191601

**Published:** 2024-09-24

**Authors:** Khurshaid Anwar, Georg Thaller, Mohammed Saeed-Zidane

**Affiliations:** Molecular Genetics Group, Institute of Animal Breeding and Husbandry, Christian-Albrechts-University Kiel, 24118 Kiel, Germany

**Keywords:** stress, bovine, sperm, *NRF2*, microsatellite, antioxidants

## Abstract

Nuclear factor (erythroid-derived 2)-like 2 (*NRF2*) is a transcription factor protein-coding gene, considered a master regulator of the cellular stress response. The genetic variations of the *NRF2* could influence its transcriptional profile and, subsequently, the stress resilience in all cell types, including sperm cells. Therefore, the sperm-borne antioxidants abundance in association with the genetic variation of a GCC microsatellite located at the 5′ upstream region of the *NRF2* gene was investigated in young (*n* = 8) and old (*n* = 8) Holstein bulls’ sperm cells at different seasons. The sperm DNA was sequenced using Sanger sequencing, while- the sperm-borne mRNA analysis was carried out using the synthesized cDNA and qPCR. The data were statistically analyzed using GraphPad Prism 10.0.2 software. The results showed that two bulls had a heterozygous genotype of eight and nine GCC repeats, while biallelic of eight, nine, and fifteen repeats were identified in two, ten, and two bulls, respectively. The computational in silico analysis revealed that the *NRF2* upstream sequence with 15, 9, and 8 GCC repeats bound with 725, 709, and 707 DNA-binding transcription factor proteins, respectively. Lower quality of sperm DNA was detected in the spring season compared to other seasons and in young bulls compared to old ones, particularly in the summer and autumn seasons. The mRNA expression analysis revealed that the *PRDX1* gene was the abundant transcript among the studied sperm-borne antioxidants and was significantly determined in old bulls’ spermatozoa. Moreover, two transcripts of the *NRF2* gene and antioxidant (*SOD1*, *CAT*, *GPX1*, *TXN1*, *NQO1*) genes displayed differential expression patterns between the age groups across seasons in an antioxidant-dependent manner. The bulls with a heterozygous GCC sequence exhibited elevated sperm-borne mRNA levels of *NRF2* and *PRDX1* transcripts. Taken together, the findings suggest that the *NRF2*-GCC microsatellite may contribute to the transcription regulation of *NRF2* transcripts and their subsequent downstream antioxidants in bovine sperm cells.

## 1. Introduction

A bull contributes half of the genetic material for the new offspring; accordingly, bull fertility is one of the main traits in cattle selection and breeding programs. In dairy cattle, milk production and reproduction traits are negatively correlated [1]. Reproductive deficiency is considered a major source of economic reduction in dairy farming [2,3]. There are different types of reproductive efficiency in dairy cattle, including infertility, low conception rate, early embryonic loss, pregnancy loss, stillbirth, calving difficulties, etc. [2]. The fertilization success and preimplantation embryo development mainly rely on the genetic and epigenetic contributions of both bull (sperm) and cow (oocyte) [4]. Bull contribution has a vital impact on reproduction efficiency loss [4]. However, there is often an underestimation of the bull’s negative influences on fertilization success, embryo quality, development, and pregnancy success. It was shown that there is a positive correlation between embryo mortality losses and low-fertility bulls compared to high-fertility bulls [5,6]. In this regard, several seminal traits, including abnormalities of the sperm cells in terms of morphology, abnormalities of sperm membranes such as live-dead count, targeted fluorochromes, acrosome qualities, and mitochondrial membrane potentials are the common markers developed to detect these embryonic failures [4]. Moreover, the reactive oxygen species (ROS) are also good indicators of sperm quality under stress conditions [7]. Oxidative stress, stemming from an imbalance between reactive oxygen species (ROS) and the antioxidants, ultimately diminishes both male and female fertility [8,9]. ROS is a term used for various molecular oxygen derivatives that are produced in live cells as normal attributes and byproducts of aerobic conditions [10]. The balanced levels of ROS serve useful roles in regulating cellular biological functions [11]. Numerous comprehensive studies demonstrated that the constant productive and reproductive stresses in cattle resulted in excess amounts of ROS which led to dysregulation of physiological homeostasis in different reproductive cell types [12].

Stress is a main factor in reproductive losses [13]; for instance, heat exposure to 40 °C increases the percentage of abnormal sperm cells and decreases the number of live sperm cells [14]. Stress has several negative impacts on the morphology and cellular components, including the DNA of the sperm cells in the bovine [14]. Although there is a slight impact of mild and moderate stress on sperm morphology and the number [15]. , it was also concluded that the spermatozoa with sperm chromatin aberration could have normal morphology [16]. Furthermore, the impact of stress on semen quality is high in young bulls [17]. Particularly, a study showed that young Holstein bulls (up to 30 months old) are more affected by heat stress compared to mature bulls (4 to 6 years old) in terms of sperm quality [17]. In particular, sperm concentration and motility are better in winter compared to summer [18]. Regardless of the animal’s age, a contradictory study is also present where it was noted that in winter, sperm production is reduced, and overall quality is impaired compared to the summer [19]. In addition, the season may influence the DNA regulation and function. Summer was found to be associated with high DNA fragmentation in porcine spermatozoa and subsequently reduces fertility [20].

Mammalian cells respond to stress, particularly oxidative stress, through various molecular signaling pathways, including NRF2 signaling [21]. NF-E2-related factor 2 (*NRF2*) is a transcription factor protein-coding gene that is considered a key master transcription regulator of numerous antioxidant enzymes [22]. Nuclear factor erythroid 2-related factor 2 (NF-E2-related factor 2 or *NRF2*) is a transcriptional factor protein-coding gene that regulates the cellular antioxidant systems [23]. NRF2 belongs to the Cap’n’Collar (CNC) subfamily of leucine zipper (bZIP) transcription factor proteins, including NRF1, NRF2, and NRF3 [24]. The Keap1–NRF2 system is the major player in oxidative response through the regulation of the antioxidant protein-coding genes [25]. Under normal conditions, the NRF2 protein remains in the cytoplasm bound to a redox-sensitive ubiquitin protein, E3 ubiquitin ligase Keap1 (Kelch ECH-associating protein 1) [24]. The ubiquitinated NRF2 is degraded by the 26S proteasome, and the cell maintains the transcription level of the stress response gene at the basal level [26,27]. Under oxidative stress conditions, NRF2 protein is released from Keap1 protein and then translocated into the nucleus. Inside the nucleus, it transactivates a member protein of the bZIP-type transcription factor family, a small musculoaponeurotic fibrosarcoma oncogene homolog (small Maf) through heterodimer formation [28,29,30]. The NRF2-Maf complex binds to antioxidant response elements (ARE) of the DNA and initiates the transcription of the downstream antioxidant genes such as the glutathione peroxidase (*GPX1*), superoxide dismutase (*SOD1*), peroxiredoxin (*PRDX1*), glutathione S-transferases (*GST*), catalase (*CAT*), glutathione reductase (*GSR*), and thioredoxin reductase (*TRX*) [28,31,32,33,34,35,36]. Bovine *NRF2* is located at chromosome number 2 according to Ensembl genome browser 112, in which four transcripts, including *Nrf-201*, *Nrf-202*, *Nrf-203*, and *NRF2-204*, have different exons. Numerous studies demonstrated elevated expression levels of the *NRF2* gene under different conditions associated with direct cellular response to stress [37,38,39]. Furthermore, an in vivo study on dairy cattle fed with chestnut tannins [40] or rumen-protected methionine [41] showed an improvement in animals’ antioxidant response through the upregulation of putative canonical *NRF2* downstream antioxidant- and cell survival-related genes.

Genetic polymorphisms, including insertions and deletions (INDELs) in the non-coding sequence of a gene, influence transcription and splicing processes [42]. Subsequently, INDELs found near the transcription binding sites (TFBS) or within the promoter sequence have significant impacts on the expression of the gene, consequently affecting the expression level of the downstream genes. The considerable amount of genetic variation that is caused by these small INDELs is substantial. The number of INDELs represents the second most frequent type of genetic variation and has a profound functional impact on evolution, adaptation, and disease susceptibility [43]. Additionally, small INDEL variations play a prominent role in human traits, personalized medicine, and diseases [44,45]. Microsatellites (MSs) are ideal genetic markers that are tracts of variable-length repeats of short DNA sequences that exhibit high rates of mutation in the form of INDELs in repeated sequences [46]. Microsatellite mutation rates in human male germ lines range from 0 to 7 × 10^3^ per locus per gamete per generation, which is five to six times higher than that in female germ lines [47]. Recent advances in science and research have conclusively shown that insertions or deletions (INDELs) increase mutation rates either directly [48,49,50] or indirectly [51,52] and locally suppress crossovers [53]. One of the fundamental conclusions of evolutionary genetics is that the distribution of nucleotide substitutions in the genomes of living organisms is not random and is associated with phenotypic variation between individuals [54]. There was a detection of 3194 high-impact disruptive single nucleotide polymorphisms (SNP) and 745 INDELs (in 275 genes) that may affect economic dairy and beef traits [54]. A clear understanding and determination of the regulatory mechanisms involved in the activity and abundance of key genes related to stress responses will improve our strategies for breeding and selection for stress tolerance. So far, the genetic and epigenetic regulation of the bovine *NRF2* gene remains elusive. Therefore, here, we attempted to elucidate the subsequent effects of genetic variations in the identified microsatellite located upstream of the non-coding sequence of the *NRF2* gene on the antioxidant capacity of bovine spermatozoa. Furthermore, the association between antioxidant capacity and sperm quality in different seasons was examined.

## 2. Materials and Methods

### 2.1. Experimental Design

To achieve our objectives, this study was conducted on bovine semen collected and cryopreserved at the RSH (Rinderzucht Schleswig-Holstein, Neumünster, Germany) station. Semen samples were collected in the spring season from 16 Holstein bulls, including old (*n* = 8, age group 3–4 years) and young (*n* = 8, age group 1–2 years) bulls. However, based on the availability of the bulls in the station, from the 16 bulls, semen was collected from 4 old and 4 young bulls during the summer, autumn, and winter seasons. Accordingly, DNA was extracted from the sperm cells of the 16 bulls and subjected to genotype analysis of the GCC microsatellite located in the 5′ upstream region of the *NRF2* gene using Sanger sequencing. Meanwhile, semen collected during the four seasons of the year from the eight selected bulls was subjected to sperm cell DNA and RNA extraction. Thereafter, DNA fragmentation analysis was performed. In parallel, the RNA was subjected to expression analysis of sperm-borne NRF2 signaling-related genes, including three different transcripts (201, 202, and 203) of the *NRF2* gene and the downstream antioxidant genes (*PRDX1*, *SOD1*, *GPX1*, *CAT*, *NQO1*, and *TXN1*). Additionally, based on the genotyping results, an in silico analysis was performed using different sequence lengths of the candidate microsatellite (GCC) to predict changes in the transcription activity of the *NRF2* gene.

### 2.2. Sperm DNA Extraction and DNA Fragmentation Analysis

DNA was extracted from sperm cells (one straw per animal) using a manual protocol. For that, sperm samples were thawed at 37 °C and then centrifuged at 13,000 rpm for 10 s. Thereafter, the pellets were washed twice with phosphate buffer saline (PBS) and incubated overnight at 65 °C in a lysis buffer containing 100 mM NaCl, 10 mM Tris-base pH 8.2, 2 mM EDTA pH 8.2, 0.5 M DTT, 10% SDS, and proteinase K. The lysate was then mixed with 6 M NaCl (*V*/*V*), followed by centrifugation at 13,000 rpm for 13 min. The clear supernatant was mixed with 100% ethanol and centrifuged at 13,000 rpm for 1 min. Thereafter, the pellets were dissolved at 37 °C in Tris EDTA buffer. The concentration of the extracted DNA was measured using a NanoDrop 1000 spectrophotometer (PEQLAB Biotechnologie GmbH, Erlangen, Germany). Furthermore, the integrity of intact and fragmented DNA was assessed on a 1% agarose gel run at 80 voltages for 1 h. Finally, images were documented using a gel documentation device (Bio-Rad Laboratories Inc., Hercules, CA, USA).

### 2.3. Conventional PCR and Sanger Sequencing

The DNA sequence repeat variation in the GCC microsatellite located at the 5′ upstream region of the *NRF2* gene was analyzed using Sanger sequencing. For that, the extracted DNA from each bull along with the forward 5′CGGAAGGGCAAACTGAGAGT3′ and reverse 5′GCAGCTCCAAGTCCATCATG3′ strand primers designed for the 5′ upstream sequences, including the GCC repeats, were used for polymerase chain reaction (PCR) amplification. The samples were incubated in a PCR cycler adjusted for 5 min at 95 °C, followed by 39 cycles of 95 °C for 10 s, 55 °C for 20 s, and 72 °C for 30 s. Thereafter, a part of the PCR product (437 bp) from each sample was loaded on a 2% agarose gel and subjected to electrophoresis for 30 min at 120 voltages. Accordingly, 7 µL of the PCR products were purified using ExoSAP IT kits (Thermofisher Scientific, Darmstadt, Germany) according to the manufacturer’s instructions. The purified products were then sequenced using the forward and reverse primers separately using the Applied Biosystems Hitachi ABI Prism 3130xl Genetic Analyzer (Applied Biosystems, Foster City, CA, USA). The sequence data were analyzed for GCC repeat length differences as well as the heterozygosity or homozygosity using Sequencher 5.0 software.

### 2.4. RNA Extraction and cDNA Synthesis

Sperm-borne RNA was extracted using the RNeasy Mini Kit (Qiagen, Hilden, Germany) according to the manufacturer’s instructions, with some modifications. Briefly, sperm cells were incubated with Qiazol buffer in addition to beta-mercaptoethanol for 15 min, following the kit manufacturer’s instructions. After the washing steps, the column was incubated with 50 µL elution buffer for RNA elution. A NanoDrop 1000 Spectrophotometer (PEQLAB Biotechnologie GmbH, Erlangen, Germany) was used to measure the RNA concentration. Thereafter, RNA at a concentration of 120 ng was standardized using RNAse-free water for cDNA synthesis. cDNA was synthesized by reverse transcription using the first strand cDNA synthesis kit (Thermofisher Scientific, Darmstadt, Germany), following the manufacturer’s instructions.

### 2.5. Sperm-Borne mRNA Expression Analysis

The mRNA levels of sperm-borne NRF2 signaling-related genes, including *KEAP1*, *NRF2* transcripts (*NRF2-201*, *NRF2-202*, and *NRF2-203*), *PRDX1*, *GPX1*, *CAT*, *SOD1*, *NQO1*, *TXN1*, and *NRF1*, were determined using the primers listed in Table 1, designed for each gene using the Primer3 online tool version 4.1.0. The quantitative PCR (qPCR) was performed using synthesized cDNA and iTaqTM Universal SYBR^®^ Green Supermix (Bio-Rad Laboratories GmbH, Feldkirchen, Germany). A total reaction volume of 20 μL was run using the following program: 95 °C for 3 min (1 cycle), followed by incubation at 95 °C for 15 s, then at 60 °C for 45 s (45 cycles), and finally, a melting curve was performed. Relative mRNA expression analysis was performed using the delta-delta CT (2^−ΔΔCT^) method with *GAPDH* and *B2M* genes as endogenous normalizers.

### 2.6. In Silico Prediction and Identification of DNA-Binding Proteins along with Different GCC Repeat Lengths

To identify the consequences of different genotypes of the GCC microsatellite on the transcriptional regulation of the bovine *NRF2* gene, the online in silico analysis tools named PROMO, v3.0.2 (https://alggen.lsi.upc.es/cgi-bin/promo_v3/promo/promoinit.cgi?dirDB=TF_8.3, accessed on 26 January 2024) and MEME Suite 5.5.4 (https://meme-suite.org/meme, accessed on 26 January 2024) were applied to predict the number and type of DNA-binding transcription factor proteins. The TRANSFAC database was utilized, and 214 transcription factors were considered to detect unique motifs in the *NRF2* gene, accommodating variations in GCC repeat insertions or deletions. Accordingly, the DNA sequences obtained from Sanger sequencing for the *NRF2* 5′ upstream region, including different numbers (8x, 9x, and 15x) of the GCC microsatellite, were uploaded as the query sequences. A list along with a schematic image of probable transcription factors binding to the same DNA sequence was developed. Various anticipated parameters were determined for each transcription factor DNA-binding position. Furthermore, the transcription factors were listed, in parenthesis, with the database accession number; the start and end positions of the potential DNA-binding sequences along with the dissimilarity percentage, in addition to the nucleotide sequence of the potential binding site, and the random expectation (RE), which indicates the anticipated occurrences of the match in a random sequence with the same length as the query sequence and is presented as both equiprobability and probability.

### 2.7. Statistical Analysis

The mRNA expression data are presented as the geometric mean ± geometric standard deviation (GM ± GSD). Statistical analyses were performed using the GraphPad Prism 10.0.2 software. Statistical differences between the four different seasons within the same age group of the bulls were analyzed using one-way ANOVA followed by a multi-comparison Tukey test. However, statistical differences between young and old bulls within each season were analyzed using a Student’s *t*-test. Statistical differences were considered significant at *p* ≤ 0.05.

## 3. Results

### 3.1. Differential DNA Sequence Length of the GCC Microsatellite Located in the 5′ Upstream of the NRF2 Gene

The amplified PCR product (437 nucleotides), including the candidate microsatellite located in the 5′ upstream region of the *NRF2* gene, was genotyped for the studied 16 Holstein bulls. While the NCBI reference genome data of nine repeats of the GCC microsatellite (9x) were identified in the DNA sequence of 10 Holstein bulls in our experiment, we also identified a polymorphic number of GCC repeats. A biallelic insertion of six GCC repeats (15x) was detected in the DNA sequence of two bulls. However, two other bulls showed a biallelic deletion of one GCC repeat (8x) in the DNA sequence of the target region. Additionally, analysis of the DNA from two more bulls showed a single deletion of one GCC repeat in one of the parental strands, resulting in a heterozygous sequence with one allele featuring nine GCC repeats, as shown in Table 2.

### 3.2. Various Repeats of the GCC Microsatellite Influenced the NRF2 DNA-Binding Proteins

**Figure 1:**
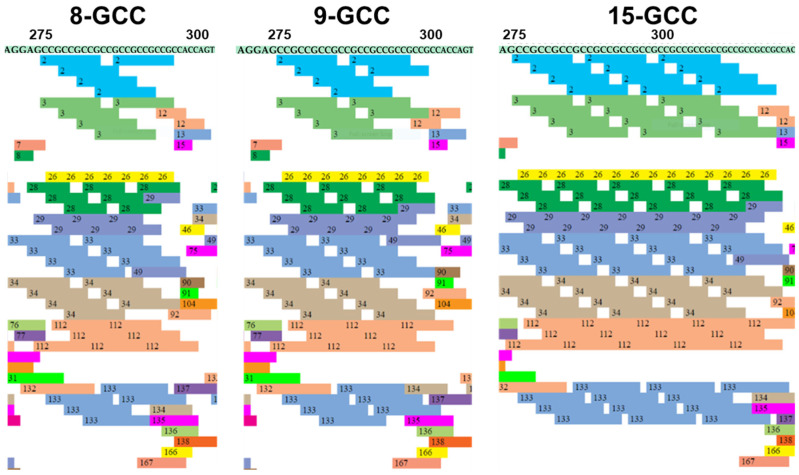
Transcription factors binding to sequences included the GCC microsatellite located in the 5′ upstream region of the *NRF2* gene. Different colors represent different transcription factor proteins.

In this analysis, we identified potential DNA-binding sites for transcription factor proteins using PROMO software v3.0.2, which relies on the TRANSFAC v8.3 database. A list of transcription factors in colored boxes (Table 3) identified to bind to a specific sequence in the region of interest. Furthermore, the positional binding patterns of TF motifs showed striking differences among 8, 9, and 15 GCC microsatellite repeats. The DNA sequences with 15x GCC, 9x GCC, and 8x GCC repeats showed a binding affinity for 214 transcription factors (Figure 1 and Table 3). Additionally, these TF bound 725, 709, and 707 times to *NRF2* DNA sequences with six GCC insertions, wild type, and one GCC deletion. Factors predicted in three input sequences within a dissimilarity margin of less than 15% for successfully obtaining many known TF–INDELs associations (Table 3).

We identified regulatory transcription factors i.e., WT1 I-KTS, E2F-1, DRF1.3, Sp1, ZF5, Sp1, NRF2, JunD, and Elk-1, for the *NRF2* gene. These transcription factors showed similar dissimilarities and the lowest relative entropy (RE) for 8, 9, and 15X GCC repeats, suggesting their higher affinity and specificity. Furthermore, our sequence of interest, which had 8, 9, and 15 GCC repeats, exhibited binding sites for a total of 214 transcription factors. Moreover, these transcription factors were selected based on relative entropy equally (RE equally), relative entropy query (RE query), string, start and end positions, and dissimilarity statistics (Table 4).

**Figure 2:**
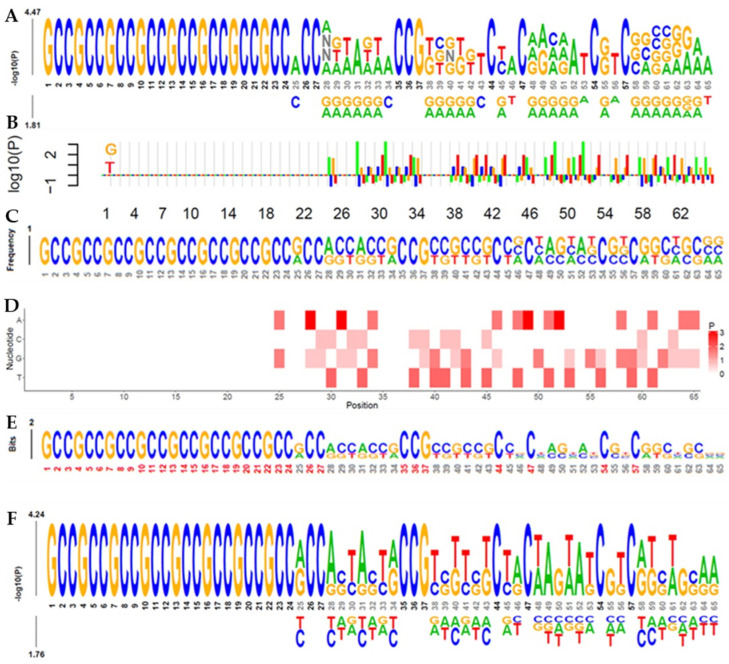
(**A**) k-mer logo generated by kpLogo using 65 nt, with k ranging from 1 to 4, allowing degenerate nucleotide N. (**B**) Comparison of a barplot (left) and a kpLogo probability logo plot (right) for depicting the *p*-values from Mann–Whitney U tests. (**C**) The abundance of k-mers, (**D**) Heatmap probability logos generated by kpLogo. (**E**) The information content (entropy) for 65 k-mers was measured in bits, with sequence logos showing the frequencies scaled relative to the information content (a measure of conservation) at each position. (**F**) In the probability logos, residues are scaled relative to the statistical significance (−log10(*p*-value)) of each residue at each position.

The 65-mer sequences are color-coded: yellow and blue with no insertion. Yellow, blue, red, and green mers show the insertion and SNPs. Enriched residues are stacked at the top, whereas depleted residues are stacked at the bottom. Nucleotide positions with significant relevance (Bonferroni corrected *p* < 0.01) are highlighted (red coordinates), as are the fixed positions of the GG PAM (black coordinates). Moreover, the sequence logo of *NRF2* 5′ upstream region is represented by a stack of bases in Figure 2, the overall height of each stack being proportional to the sequence conservation at that place, measured in bits, and the height of each base is proportional to the observed frequency of the associated nucleotide. All stack bases are arranged from most to least frequent. We identified a conserved GCC microsatellite in the sequence of interest from positions 1 to 24 in Figure 2A–F. Following the conserved GCC microsatellite, the 5′ upstream region exhibits highly predictive variability. The color coding of the residues is based on their chemical characteristics Top of Form (Figure 2).

To identify important k-mers, Sanger-sequenced PCR products of base pairs 434, 437, and 455 were used. Utilizing transcription factor binding motif position weight matrices (PWMS) or position-specific score matrices (PSSMS), we identified 27 important k-mers spanning from single nucleotides to groups of four nucleotides. Their location, shift, statistical measures, *p*-value, corrected *p*-value, observed and expected fractions, ratios, and local importance were among the many factors that were taken into account to obtain these results (Table 5).

**Figure 3:**
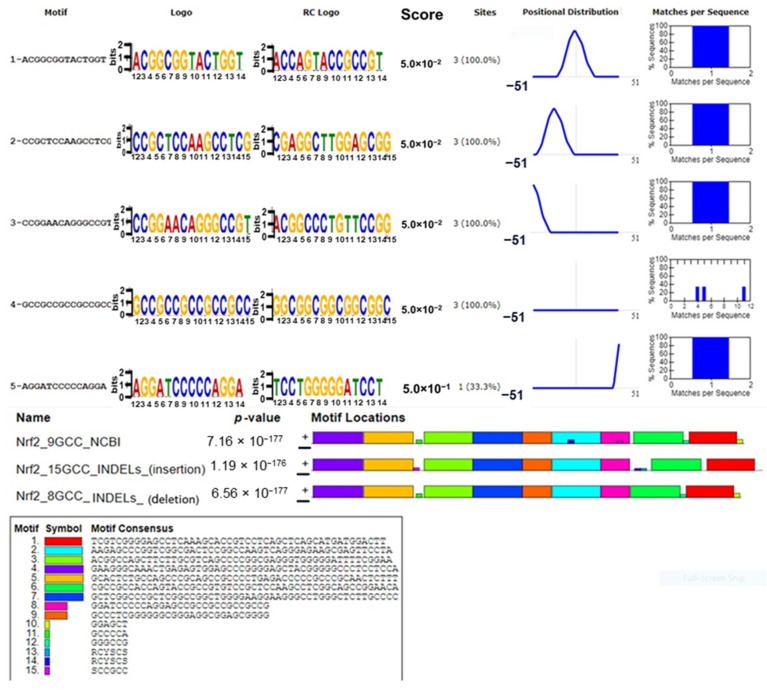
XSTREME: Comprehensive analysis of 15 motifs of *NRF2* gene sequences clustered by similarity and ordered by E-value.

Out of the fifteen identified motifs, nine large motifs and three micro motifs with variations in adenine or guanine (R), cytosine or thymine (Y), and guanine or cytosine (S) nucleotides were found based on score, sites, positional distribution, and matches per sequence with a *p*-value of 1.19 × 10^−176^ in sequence with 6 GCC repeats insertion. In addition, we predicted three minor and nine major motifs in the sequence with one GCC deletion (*p*-value = 6.56 × 10^−177^). With a *p*-value of 7.16 × 10^−177^, the wild-type sequence displayed nine major motifs and variants in the R, Y, and S nucleotides in five short motifs. The *NRF2* upstream region exhibited five conserved motifs, each represented by a sequence logo and its reverse-complement logo. The first four motifs shared similar alignment qualities, significance, and motif scores. However, we identified the motif AGGATCCCCAGGA with a higher score of 5.0 × 10^−1^ (0.5), suggesting its distinctive importance. The specific locations of sites for the first four motifs are consistent, while the fifth motif diverges due to its proximity to insertion and deletion sites (Figure 3).

Analyses of the motifs further revealed different positional distributions for all motifs; motif 4 exhibited a unique 38% percent sequence coverage, with matches per sequence at 4, 5, and 11 on the *x*-axis. Variability of purine (R, G, or A), pyrimidine (Y, T, or C), and guanine/cytosine (S, G, or C) in one motif was found for the sequence with insertion of the GCC repeat, while no variability was found for the sequence with GCC nucleotide deletion. These variable nucleotides (R, Y, and S) in all small motifs are situated on the minus strand.

### 3.3. Various Repeats of the GCC Microsatellite Associated with Different Levels of Sperm-Borne NRF2 Transcripts

The eight bulls used in the mRNA analysis showed a homozygous genotype of 9x GCC in five bulls, including four young bulls and one old bull. Additionally, one old bull showed a heterozygous genotype of 8/9x GCC, and two old bulls showed homozygous 8x GCC repeats (Table 2). Generally, sperm-borne mRNA expression analysis revealed high levels of *NRF2* transcripts and downstream antioxidants in old bulls compared to young bulls in a season-dependent manner. However, the heterogeneous old bull) within the old bulls group showed lower delta Ct values, indicating higher mRNA levels for the *NRF2-201* transcript and downstream antioxidants (Supplementary Figure S1).

### 3.4. Season and Age Group Influenced Sperm-Borne mRNA of the Antioxidants

**Figure 4:**
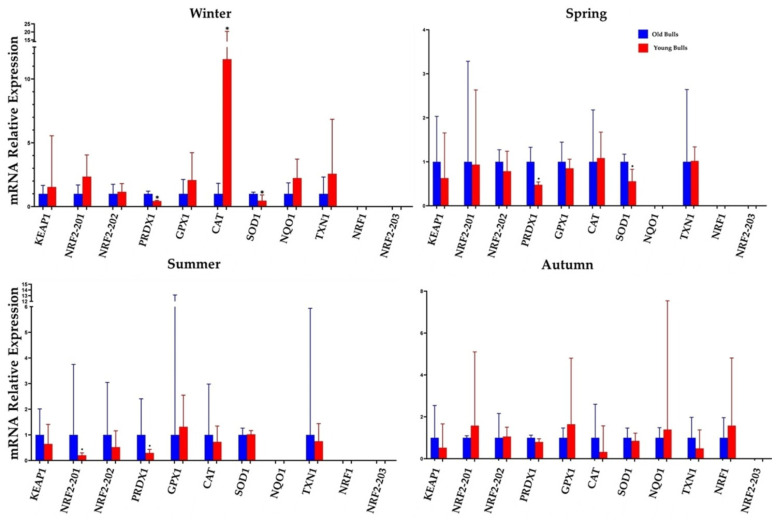
Sperm-borne mRNA levels of oxidative stress-related genes in winter, spring, summer, and autumn seasons. Kelch Like ECH Associated Protein, Nuclear Factor Erythroid 2-Related Factor 2, Peroxiredoxin-1, Glutathione Peroxidase 1, Catalase, Superoxide Dismutase 1, NAD(P)H Quinone Dehydrogenase 1, Thioredoxin and Nuclear Respiratory Factor 1. Values are presented as geometric mean ± SD. * *p* < 0.05, significant difference.

**Figure 5:**
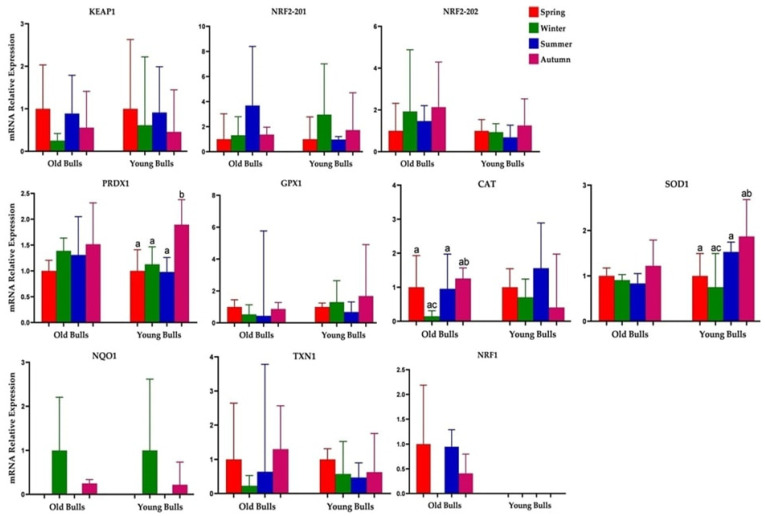
Differential expression patterns of sperm-borne antioxidants within different seasonal groups. Kelch Like ECH Associated Protein, Nuclear Factor Erythroid 2-Related Factor 2, Peroxiredoxin-1, Glutathione Peroxidase 1, Catalase, Superoxide Dismutase 1, NAD(P)H Quinone Dehydrogenase 1, Thioredoxin and Nuclear Respiratory Factor 1. Values are presented as geometric mean ± geometric SD. a, b, ab, and ac *p* < 0.05; significant difference.

The mRNA expression patterns of several genes related to antioxidant and stress response mechanisms were investigated in the sperm of both old and young bulls across different seasons. There was a season-dependent differentiation in the sperm-borne mRNA levels of the different investigated stress-related transcripts within the young and old bull groups. In old bulls, compared to their young counterparts, *PRDX1* and *SOD1* mRNA showed significantly increased expression in the winter and spring seasons (Figure 4). Moreover, *NRF2-201* and *PRDX1* displayed an upregulation in the spermatozoa of old bulls in summer (Figure 4). *GPX1* mRNA exhibited a non-significant high level in autumn and winter in young bulls. However, *CAT* showed a significantly high level in winter for young bulls (Figure 4). *NQO1* was not detected in spring and summer in both old and young bulls, with similar expression patterns observed in winter and autumn (Figure 4 and Figure 5). However, the sperm-borne mRNA of the *NRF1* gene was detected only in the sperm cells of young bulls in autumn (Figure 4 and Figure 5).

### 3.5. Substantial Seasonal Variability in Oxidative Stress-Induced Signature Genes

We compared gene expression differences between old and young bulls across the four seasons. Ten oxidative stress-related genes in the current study exhibited differential sperm-borne mRNA levels in an age-dependent manner. For example, *PRDX1* and *SOD1* were differentially expressed in young bulls in the autumn season compared to other seasons. The mRNA of sperm-borne *CAT* in old bulls showed a lower level in winter compared with the other seasons. *NQO1* was not detected in the spring and summer of both young and old bulls’ spermatozoa, while *NRF1* was detected in the spring, summer, and autumn of old bulls only (Figure 5).

### 3.6. Higher Sperm DNA Integrity of Old Bulls under Seasonal Stress and Detection of DNA Damage by Gel Electrophoresis

**Figure 6:**
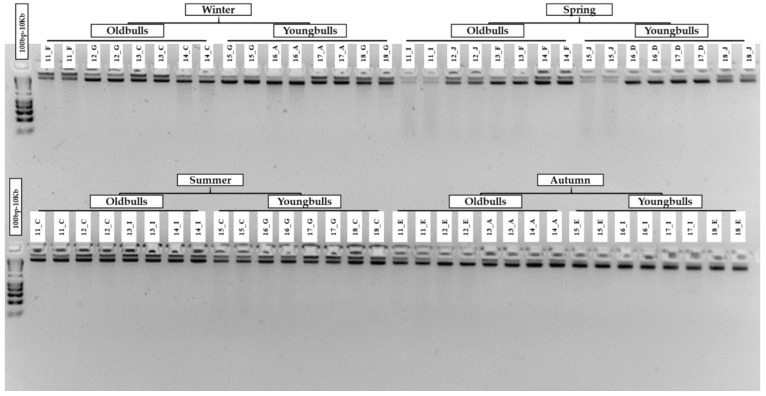
Seasonal comparison of gDNA fragmentation in the spermatozoa of young and old Holstein bulls across different seasons in duplicate for each bull, as visualized by agarose gel electrophoresis. Numbers 11–14 depict old bulls, and 15–18 depict young bulls. Upper lanes (11–18) display gDNA fragmentation in winter and spring. Bottom lanes (11–18) display gDNA fragmentation in summer and autumn. The first lane in the top and bottom rows corresponds to the 100 bp–10 kb DNA ladder.

The impact of semen service time and season on sperm DNA was examined for old and young bulls (Figure 6). A total of eight Holstein bulls (young *n* = 4, old *n* = 4) were subjected to agarose gel electrophoresis of DNA extracted from sperm cells of old and young bulls. In spring, one old bull (labeled 11_I) showed a higher level of reversible DNA fragmentation. Old bulls manifested minimal to no fragmentation in summer and autumn. One young bull (labeled 15_J) also experienced complete reversibility of DNA fragmentation in the spring season, with no significant fragmentation in the summer and autumn seasons. Except for one old and young bull in spring, no DNA smearing was observed in agarose gel electrophoresis, indicating its high quality and verification through subsequent molecular applications. Minimal smearing was observed in the gDNA of young bulls during summer and winter, with an overall good quality of gDNA in subsequent molecular techniques and procedures.

## 4. Discussion

Oxidative stress has detrimental effects on several reproductive processes, including spermatogenesis, folliculogenesis, fertilization, preimplantation embryo development, and pregnancy success [55]. Reactive oxygen species (ROS) are naturally produced as byproducts of sperm metabolism [56]. However, male gonads lacking defense and protective mechanisms against oxidative stress are vulnerable to infertility consequences [57,58]. Balanced concentrations of ROS are necessary for the fertilizing ability of sperm cells, while excessive amounts reduce the fertilization rate [59,60]. Despite exogenous factors, spermatozoa are exposed to stress during spermatogenesis, epididymis storage, ejaculation, cryopreservation, and the journey in the female reproductive tract. Sperm cells are among the cells susceptible to oxidative stress, and they lack antioxidant capacity due to their limited cytoplasm that enables an appropriate number and amounts of defensive enzymes [61]. The increment in ROS production leads to lipid peroxidation [62], which in turn results in the loss of plasma membrane integrity, mitochondrial membrane dysfunction, chromatin degradation [63,64], and morphological and motile abnormalities in sperm cells. Moreover, High ROS levels in spermatozoa can lead to direct DNA damage [63,64,65].

Mammalian cells respond to stress by utilizing a range of multiple genetic and epigenetic regulatory mechanisms and signaling. The bovine *NRF2* gene is a member of a small family of basic leucine zipper (bZIP) proteins. It plays various cellular roles in regulating redox homeostasis, mitochondrial function, oxidative phosphorylation, ATP production, and fatty acid oxidation [66]. Numerous studies have shown the role of *NRF2* in the oxidative stress response. Under stress, the transcription factor NRF2 separates from KEAP1 moves into the nucleus and binds to the DNA sequence of the antioxidant response elements to initiate the expression of a wide range of genes that code for antioxidant proteins [31,32,33,67,68].

However, the genetic markers associated with the transcription activity and capacity of *NRF2* and its downstream antioxidants in bovine spermatozoa are still unknown. Therefore, the current study aimed to investigate the genetic regulation of the *NRF2* gene and its activity association with spermatozoa antioxidant capacity in young and old Holstein bulls under different seasons. We found that the DNA sequence of the 5′ upstream untranslated region of the bovine *NRF2* gene contains a microsatellite sequence motif with nine GCC repeats. Accordingly, we hypothesized that this microsatellite is a part of the promoter region, which may genetically or epigenetically regulate the transcription activity of the *NRF2* gene and the subsequent downstream antioxidant genes. It may also influence the splicing process of *NRF2* pre-mRNA transcripts, particularly the *NRF2-201* transcript. Mutation of the *NRF2* gene alters the interaction of KEAP1 and NRF2 protein residues, thus triggering the activation of many Basic leucine zipper (bZIP) domains of Cap’n’Collar (CNC) transcription factors [48]. The most common genetic variation classes linked to diseases and other traits were shown to be 86% 1–5 bases long INDELs, 14% tandem repeats [69], and INDELs 1 to 10 bases long in chickens and humans [70,71,72]. Although other studies strongly connected SNPs in the *NRF2* gene to better performance [73], animals with a heterozygous GCC deletion in our study showed lower Ct values (high abundance) for all downstream analyses with relatively low gDNA fragmentation and a better response to oxidative stress of spermatogenesis in old bulls. Our results suggest that *NRF2*_(GCC)n gene insertion and deletion influence susceptibility to oxidative stress, as young bulls in our study showed no deletion of GCC, proving a close association of INDELs with low response to oxidative stress. Data on the expression of downstream genes in both young and old bulls with 15x GCC are currently lacking in our study, as we selected only four young and old bulls for gene expression across the year from the eight genotyped bulls in each group. Many previous investigations have assessed the effects of INDELs as temporary and can be lost during the course of evolution, thus influencing the diversity of genetic traits within a population [49,50,52,53,74]. In contrast, other studies assessed the connection between gene functions with INDELs epistatically, explaining the genetic hitchhiking of the co-occurring variants [75]. We report here that sequence variation in the *NRF2* DNA sequence causes a high-affinity to a low-affinity binding site. We identified additional determinants of the specificity of many transcription factors (TFs). Our analysis of 214 pairs of full-length TFs showed 18 times more affinity for sequences with 18 nucleotides (6x GCC) insertions compared to sequences containing three nucleotides (1x GCC) deletions. These bZIP transcription factors may share the same single binding site; the string CCGCCGCCGC is shared by nine transcription factors, and this intricate regulation of the target gene is made possible by the tiling arrangement of the transcription factors. Most of the 1–5 bp INDELs have a much smaller effect on TF binding, while other investigations have stated that SNPs and INDLs can accurately disrupt TF binding sites by altering motifs and subsequently influencing gene expression [31,32,33,76,77].

In our analysis, we found no differential effects of INDELs on dissimilarity and string length, as transcription factors WT1, ZF5, E2F-1, SP1, NRF2-MafK, JunD, UME6, and Elk-1 displayed the same dissimilarity and binding string length of 10, 3, 7, 9, 7, 7, 11, and 5 nucleotides for the 15x, 9x, and 8x GCC repeats. On the other hand, the INDEL was found to significantly alter the query and the string entropy. The sequence-specific transcription factors bind to similar sequences, except for the ELK1 protein, with differences in the specificity of the TF [78,79]. The later findings are not consistent with our results, in which ELK-1 binds to only the same length of GCC nucleotides in the sequence of the *NRF2* gene, which might be due to the string being located in the 5′ upstream region of the gene. Additionally, in the present study, CREMtau2, ZF5, E2F-1, f(alpha)-f(epsilon), DRF1.1, DRF1.3, Sp1, and WT1 showed specificity and high binding affinity for the highly conserved repeated GCC region in the 5’ upstream region of the *NRF2* gene in all sequences with INDELs, providing enough information about their role in activating the *NRF2* gene. Furthermore, similar to the 24-mer conserved region, *NRF2* 5’ upstream of the 40-mer region of the *NFATC1* gene demonstrated the highest cumulative score of binding specificity [80]. The similarity between the two results may be attributed to the abundant guanine and cytosine nucleotides in both sequences.

K-mer refers to a nucleotide sequence of a certain length, and is an important tool in many genetic studies. In the present study, we identified 27 different important k-mers, ranging in size from 1- to 4-mers. Most of these k-mers are mainly located in the candidate 5’ upstream region of the *NRF2* gene, where they might serve as regulatory motifs and binding sites for transcription factors. A previous study showed that CACGNG, CACGTT, and CATG(T/C) G are functional motifs that play an important role in transcription and post-transcriptional regulation [81]. Here, 15 significant motifs from multiple locations in the *NRF2* DNA sequence were selected, from which 14 motifs in the wild-type sequence were observed, while 12 motifs each were found in sequences with INDELs with different *p*-values of 6.56 × 10^−177^, 7.16 × 10^−177^, and 1.19 × 10^−176^ for 8x GCC, 9x GCC, and 15x GCC, respectively. Two small motifs in the sequence of 15x GCC, RCYSCS, have the potential to bind to many transcription factors. On the other hand, six adjacent motifs in the sequence of the *NRF2* gene with 8x GCC of different lengths might positively influence the activity of *NRF2* and its downstream antioxidants. Genetic variations, including INDELs, affect TF protein-DNA binding [55,82,83,84,85,86,87,88,89].

Sperm-borne mRNA expression analysis revealed that old bull spermatozoa with a 1x GCC deletion in the *NRF2* gene had a higher level of *NRF2-201* transcript. Moreover, *PRDX1* and *SOD1* were the most abundant sperm-borne antioxidants, which were significantly higher expressed in winter, spring, summer, and autumn. It has been reported that *PRDX1* and *SOD1* are present in sperm-borne mRNA under stress [90,91]. Furthermore, *NRF2-201* and *NRF2-202* were upregulated in the spermatozoa of old bulls in the spring, summer, and summer seasons. Similarly, *NRF2* downstream antioxidants are activated under oxidative stress conditions [92]. A previous study demonstrated that increased mRNA levels of *NRF2* downstream genes, such as *NQO1* and *TXN1*, were observed under acute inflammation-induced oxidative stress [93], which was confirmed in our study by the upregulation of antioxidant genes, such as *CAT*, *TXN1*, and *NQO1* in young bulls in the winter season. Catalase (CAT) is regarded as one of the most efficient enzyme systems displaying zero-order kinetics and is upregulated under cold stress [94]. This enzyme regulates the sperm maturation in the epididymis and protects sperm from oxidative stress through the breakdown of hydrogen peroxide and hydroperoxides [95]. The current findings of *CAT* antioxidant significant upregulation in younger bulls in winter may confirm its key role and further advocate the introduction of bulls to semen collection in spring or summer for the first time. In the current study season, and age-specific genes, such as *NRF1* and *NQO1*, were identified in the spermatozoa of old and young bulls. The mRNA of NRF1 is expressed in the winter season and in all seasons of old and young bull spermatozoa, respectively. *NQO1* was not detected in the spring or summer in both old and young bulls, indicating the age- and season-specific expression of these antioxidants. These results demonstrate that seasonal variation is an important environmental regulator of gene expression [96].

Expression levels of sperm-borne antioxidant marker genes in old and young bulls across the year. We observed that *KEAP1*, *PRDX1*, *GPX1*, and *NQO1* gene expression followed a similar seasonal pattern in both age groups. The upregulation of antioxidants at the transcription level in spermatozoa [97,98] led us to postulate constant and stable expression levels of *KEAP1*, *PRDX1*, *GPX1*, and *NQO1* in all groups of bulls under the same conditions, suggesting a role for these genes in the fundamental processes of sperm response to oxidative stress, highlighting the potential of these genes as stress response mRNA markers. We further identified seasonal age-related and genetic-related variations in the gene expression profiles of *NRF2* and its downstream antioxidants (Supplementary Figure S1) in the spermatozoa of young and old bulls, providing valuable insights into the understanding of these genes as genetic-sperm-borne stress markers. The mRNA abundance of these oxidative-related transcripts, including *NRF2*, *PRDX1*, *CAT*, *GPX1*, *SOD1*, *NQO1*, and *TXN1*, has been linked to spermatogenesis, intact gDNA, fertility, motility, quality, and protection of sperm cells from oxidative/nitrosative stresses [55,82,83,84,85,86,99].

In this regard, we observed high-quality gDNA in winter, summer, and autumn; however, spring revealed a reversible higher gDNA fragmentation in both young and old bulls. Evaluation of DNA damage induced by tropical summer in boar spermatozoa [74,100]. Consistent with this, our results showed that higher fragmentation in the spring season in both old and young bulls was modifiable and reversed in summer and autumn. The higher pregnancy rates in spring could be due to the low DNA fragmentation obtained in this period [101]. These findings are supported by our results, in which bulls with highly fragmented gDNA in the spring season showed the absence of three critical antioxidant genes: *NRF1*, *NRF2*, and *NRF2-203*. The results of gDNA fragmentation and its consequences on young bulls’ gene expression differ from those of [100,102], in which the authors weakly linked conventional sperm parameters to DNA fragmentation. In conclusion, this study is the first to shed light on the genetic regulation of the bovine *NRF2* gene and its consequences on sperm antioxidant capacity. The data revealed genetic variations between bulls in the DNA sequence of a microsatellite (GCC) located at the 5′ upstream region of the *NRF2* gene. The genotypes of the bulls showed 8x, 9, 8x/9x, 9x, or 15x GCC repeats. The variation in GCC length was associated with variation in the transcription factor proteins binding to the 5′ upstream nucleotide sequence of the *NRF2* gene. There was an increase in the abundance of *NRF2* transcripts and downstream antioxidants in the sperm-borne mRNA of the heterozygous bull. The *PRDX1* and *SOD1* genes were the most abundant sperm-borne-antioxidants, particularly in old bulls, in a season-dependent manner. Finally, this study is the first to investigate the genetic regulation of the *NRF2* gene associated with sperm-borne antioxidant capacity in Holstein bulls. The 8x and 8x/9x genotypes of the GCC microsatellite located at the 5′ upstream region of the *NRF2* gene are associated with higher sperm-borne antioxidant abundance. Further investigations are ongoing on the influence of different genotypes of the GCC microsatellite on the stress tolerance and sperm-related phenotypes of in vitro incubated spermatozoa, in addition to the methylation pattern of the candidate microsatellite.

## Figures and Tables

**Table 1:** Primer list used for sperm-borne mRNA analysis.

Transcript	Gene Name	Forward and Reverse Primers	Product Size
NM_001101142.1	*KEAP1*	5′TCACCAGGGAAGGATCTACG3′ II5′AGCGGCTCAACAGGTACAGT3′	199
NM_001011678.2	*NRF2-201*	5′CATGATGGACTTGGAGCTGC3′ II5′AGGCTTTCTCTTGCTCCTTT3′	209
ENSBTAT00000072818.2	*NRF2-202*	5′AAGAGTACTGGAGTGGGGTG3′ II5′CATGCTCCTTCTGTCGTTGA3′	196
NM_174076.3	*GPX1*	5′ACCCAGATGAATGACCTGCA3′ II5′GCCATTGACCTCGCACTTTT3′	189
NM_174431.1	*PRDX1*	5′TCACTTCGTCACCTGGCAT3′ II5′GCCAACAGGAAGGTCGTTTA3′	211
NM_001034034.2	*GAPDH*	5′CCCAGAATATCATCCCTGCT3′ II5′CTGCTTCACCACCTTCTTGA3′	185
NM_001035386.2	*CAT*	5′TGGGACCCAACTATCTCCAG3′ II5′AAGTGGGTCCTGTGTTCCAG3′	177
NM_001034535.1	*NQO1*	5′AACCAACAGACCAGCCAATC3′ II5′CACAGTGACCTCCCATCCTT3′	154
NM_001098002.2	*NRF1*	5′GGCAACAGGAAAGAAACGGA3′ II5′ACGCACCACATTCTCCAAAG3′	217
NM_173968.3	*TXN1*	5′AGCTGCCAAGATGGTGAAAC3′ II5′ACTCTGCAGCAACATCCTGA3′	215
NM_174615.2	*SOD1*	5′TGCCATCGTGGATATTGTAG3′ II5′GCAATTCCAATTACACCACA3′	174
NM_173893.3	*B2M*	5′TCCAGCGTCCTCCAAAGATT3′ II5′CCTTGCTGTTGGGAGTGAAC3′	222

**Table 2:** Genotyping analysis of the GCC microsatellite located in the 5′ upstream region of the *NRF2* gene.

S#	Age Groupe	Microsatellite	S#	Age Groupe	Microsatellite
1	Old bulls	9X GCC (Homozygous)	1	Young bulls	15X GCC (Homozygous)
2	Old bulls	15X GCC (Homozygous)	2	Young bulls	9X GCC (Homozygous)
3	Old bulls	8/9X GCC (Heterozygous)	3	Young bulls	9X GCC (Homozygous)
4	Old bulls	9X GCC (Homozygous)	4	Young bulls	9X GCC (Homozygous)
5	Old bulls	8X GCC (Homozygous)	5	Young bulls	9X GCC (Homozygous)
6	Old bulls	8X GCC (Homozygous)	6	Young bulls	9X GCC (Homozygous)
7	Old bulls	8/9X GCC (Heterozygous)	7	Young bulls	9X GCC (Homozygous)
8	Old bulls	9X GCC (Homozygous)	8	Young bulls	9X GCC (Homozygous)

**Table 3:** Identification of the predicted TF binding sites to specific motif sequences (colored boxes) in the regulatory 5′-upstream region, including the GCC motifs of the *NRF2* gene. # refers to transcription factor protein numbers.

#	TF	#	TF	#	TF	#	TF	#	TF	#	TF
0	DSXF [T00955]	1	DSXM [T00956]	2	WT1 I -KTS [T00900]	3	WT1 -KTS [T01839]	4	p53 [T00671]	5	ETF [T00270]
6	EIIaE-A [T00246]	7	p300 [T01427]	8	FACB [T02841]	9	R2 [T00712]	10	FOXN2 [T04206]	11	BTEB3 [T05051]
12	MF3 [T00507]	13	NF-1 [T01298]	14	AP-2alphaA [T00035]	15	Msx-1 [T02072]	16	Pax-8 [T01828]	17	Zeste [T00918]
18	Zeste [T02100]	19	C/EBP [T01386]	20	LF-A1 [T00467]	21	CREMtau [T01309]	22	CREMtau1 [T02108]	23	CREMtau2 [T02109]
24	E12 [T01786]	25	myogenin [T00528]	26	ZF5 [T02349]	27	DP-1 [T01548]	28	E2F-1 [T01542]	29	f(alpha)-f(epsilon) [T00287]
30	PEBP2alphaA1 [T01062]	31	E2F-1 [T01543]	32	Yi [T00913]	33	DRF1.1 [T05835]	34	DRF1.3 [T05837]	35	MyoD [T00526]
36	Sp1 [T00753]	37	Sp1 [T00752]	38	Sp1 [T00754]	39	Sp3 [T02338]	40	BTEB4 [T05053]	41	MYBAS1 [T05553]
42	Egr-1 [T01200]	43	Sp3 [T02453]	44	Pax-6 [T00682]	45	RC2 [T00724]	46	Zic3 [T04671]	47	TCF-2 [T01110]
48	NHP-1 [T00621]	49	E2F-1:DP-1 [T05204]	50	GCF [T00320]	51	Sp1 [T01228]	52	C/EBPbeta [T00017]	53	C/EBPalpha [T00107]
54	RFX1 [T01673]	55	MYB2 [T02536]	56	MEF1 [T00506]	57	Pu box binding factor [T00704]	58	NF-AT2 [T01945]	59	NF-AT1 [T01948]
60	TRM1 [T05311]	61	NF-AT1 [T01944]	62	HMG I(Y) [T02368]	63	STAT4 [T01577]	64	c-Ets-1 [T00112]	65	E2F [T01547]
66	E2F-5 [T01607]	67	STAT1beta [T01573]	68	E2F2 [T05976]	69	STAT6 [T01581]	70	E2F1 [T05975]	71	E2F-4 [T01546]
72	AP-2alpha [T00033]	73	TFIIB [T00818]	74	PPAR-alpha:RXR-alpha [T05221]	75	NRF2:MafK [T05666]	76	HELIOS [T06012]	77	Elk-1 [T00250]
78	C/EBPalpha [T00104]	79	EFII [T00239]	80	C/EBPalpha [T00105]	81	C/EBPalpha [T00108]	82	C/EBPbeta [T00459]	83	C/EBPbeta [T00581]
84	USF2 [T02115]	85	c-Fos [T00123]	86	Pax-2 [T01823]	87	Nkx2-1 [T00857]	88	Spz1 [T04668]	89	VDR [T00885]
90	Zic1 [T04669]	91	Zic2 [T04670]	92	USF-1 [T00875]	93	WT1 I [T01840]	94	MZF-1 [T00529]	95	COE1 [T01112]
96	NF-kappaB1 [T00593]	97	STAT5A [T04683]	98	E74A [T00208]	99	c-Ets-1 [T00111]	100	c-Ets-1 68 [T00115]	101	GAL4 [T00302]
102	NF-1 (-like proteins) [T00601]	103	SIP4 [T03600]	104	E47 [T00207]	105	CP2 [T00152]	106	PEA3 [T00684]	107	Pbx1b [T02088]
108	c-Ets-2 [T00113]	109	Elk-1 [T05013]	110	NERF-1a [T05021]	111	R1 [T00711]	112	Sp1 [T00759]	113	Sp1 [T00755]
114	Pax-5 [T01201]	115	Nkx2-1 [T00856]	116	NF-1 [T00535]	117	NF-1 [T00536]	118	NF-1 [T00538]	119	TGGCA-binding protein [T00832]
120	LIM1 [T04817]	121	AP-1 [T00029]	122	NFI/CTF [T00094]	123	EmBP-1a [T04819]	124	XPF-1 [T00906]	125	Sp3 [T02419]
126	c-Ets-2 [T01397]	127	RAR-beta:RXR-alpha [T05420]	128	COE2 [T05006]	129	MEDEA (MED) [T04379]	130	AP-2beta [T02469]	131	NF-E4 [T00560]
132	Mad [T04378]	133	WT1 [T00899]	134	Alfin1 [T04733]	135	CAC-binding protein [T00076]	136	ABI4 [T05743]	137	Pax-9a [T03593]
138	Pax-9b [T03594]	139	GAMYB [T02679]	140	YY1 [T04970]	141	AR [T00040]	142	GR [T00333]	143	HNF-3beta [T02344]
144	Elf-1 [T05012]	145	HOXA3 [T00378]	146	TAF [T00778]	147	Olf-1 [T01040]	148	TCF-1A [T00999]	149	LEF-1 [T02905]
150	TCF-4E [T02878]	151	HNF-3 [T02277]	152	DEF:GLO:SQUA [T03217]	153	TCF-3 [T02857]	154	DBP [T00183]	155	Pax-2a [T00678]
156	T3R-alpha1 [T01152]	157	DEAF-1 [T05885]	158	UME6 [T01247]	159	PR B [T00696]	160	PR A [T01661]	161	GR-alpha [T00337]
162	PR B [T00697]	163	GR-beta [T01920]	164	Adf-1 [T00008]	165	HES-1 [T01649]	166	NF-1 [T00537]	167	ENKTF-1 [T00255]
168	c-Myb [T00137]	169	PUR alpha [T05167]	170	PUR beta [T05172]	171	USF2b [T02377]	172	c-Jun [T00131]	173	JunB [T00436]
174	c-Fos [T00124]	175	c-Jun [T00132]	176	c-Jun [T00133]	177	JunD [T00437]	178	c-Fos [T00122]	179	ATF3 [T01095]
180	REB [T02808]	181	NF-1 [T00539]	182	PacC [T01679]	183	DREB1A [T05770]	184	c-Ets-1 54 [T00114]	185	MAZ [T00490]
186	AP-2 [T00034]	187	GCR1 [T00322]	188	DEF:GLO [T03216]	189	IRF-7A [T04674]	190	GT-1 [T00339]	191	POU3F1 [T00969]
192	Cutl1 [T02042]	193	AGL3 [T03025]	194	POU1F1a [T00691]	195	C/EBPdelta [T00109]	196	unc-86 [T01882]	197	MBF1 [T00492]
198	Pax-4a [T02983]	199	BR-C Z2 [T01478]	200	Dl [T00196]	201	POU3F2 [T00630]	202	DBP [T04875]	203	FOXP3 [T04280]
204	TFII-I [T00824]	205	MIG1 [T00509]	206	Pax-5 [T00070]	207	AP-1 [T01140]	208	C/EBPgamma [T00216]	209	IRF-1 [T00423]
210	IRF-3 [T04673]	211	NF-AT1 [T00550]	212	ADR1 [T00011]	213	EBF [T05427]	214	NF-X3 [T01514]		

**Table 4:** The PROMO algorithm predictions with the details of factor name, start position, end position, dissimilarity, string, RE equally, and RE query of different colors and numbers.

GCC	Factor Name	Start Position	End Position	Dissimilarity	String	RE Equally	RE Query
8	WT1 I-KTS [T00900]	277	286	14.586256	CCGCCGCCGC	0.03433	0.54917
9	WT1 I-KTS [T00900]	277	286	14.586256	CCGCCGCCGC	0.03460	0.55347
15	WT1 I-KTS [T00900]	277	286	14.586256	CCGCCGCCGC	0.03622	0.57928
8	ZF5 [T02349]	278	280	0.000000	CGC	5.98438	19.64578
9	ZF5 [T02349]	278	280	0.000000	CGC	6.03125	19.79966
15	ZF5 [T02349]	278	280	0.000000	CGC	6.31250	20.72297
8	E2F-1 [T01542]	278	284	5.331496	CGCCGCC	0.09351	1.01167
9	E2F-1 [T01542]	278	284	5.331496	CGCCGCC	0.09424	1.01959
15	E2F-1 [T01542]	278	284	5.331496	CGCCGCC	0.09863	1.06714
8	Sp1 [T00759]	282	290	10.820862	GCCGCCGCC	0.05698	0.34557
9	Sp1 [T00759]	282	290	10.820862	GCCGCCGCC	0.05743	0.34828
15	Sp1 [T00759]	282	290	10.820862	GCCGCCGCC	0.06010	0.36452
8	Nrf2:MafK [T05666]	298	304	9.333580	CCAGTAC	0.37402	0.36497
9	Nrf2:MafK [T05666]	301	307	9.333580	CCAGTAC	0.37695	0.36783
15	Nrf2:MafK [T05666]	319	325	9.333580	CCAGTAC	0.39453	0.38498
8	JunD [T00437]	235	241	6.527383	CAAGTCA	0.09351	0.02745
9	JunD [T00437]	235	241	6.527383	CAAGTCA	0.09424	0.02767
15	JunD [T00437]	235	241	6.527383	CAAGTCA	0.09863	0.02896
8	UME6 [T01247]	345	355	14.983340	GTCGTCGGGGA	0.04438	0.08094
9	UME6 [T01247]	348	358	14.983340	GTCGTCGGGGA	0.04473	0.08157
15	UME6 [T01247]	366	376	14.983340	GTCGTCGGGGA	0.04681	0.08538
8	Elk-1 [T00250]	368	372	3.960472	CGTCC	0.74805	1.25409
9	Elk-1 [T00250]	371	375	3.960472	CGTCC	0.75391	1.26391
15	Elk-1 [T00250]	389	393	3.960472	CGTCC	0.78906	1.32285

**Table 5:** The most significant k-mer at each position in our sequence of interest. Letters A, C, G, N, and T refer to adenine, cytosine, guanine, any, and thymine nucleotides, respectively.

k-mer	Position	Shift	Statistics	*p*-Value	Corrected.p	Frac.obs	Frac.exp	Obs/Exp	Local.r
A	25	0	1.51136	1.66805	−0	0.333333	0.0871795	3.82353	−9.28571
ANNA	28	0	6.21915	4.47409	−0	0.666667	0.0322581	20.6667	−2.33962
GTA	29	0	4.39941	3.12618	−0	0.333333	0.015873	21	−2.33333
TA	30	0	4.4371	3.13979	−0	0.333333	0.015625	21.3333	−2.32727
A	31	0	3.55799	3.17876	−0	0.666667	0.0871795	7.64706	−3.29114
GTA	32	0	4.39941	3.12618	−0	0.333333	0.015873	21	−2.33333
TA	33	0	4.4371	3.13979	−0	0.333333	0.015625	21.3333	−2.32727
A	34	0	1.51136	1.66805	−0	0.333333	0.0871795	3.82353	−9.28571
TG	38	0	4.4371	3.13979	−0	0.333333	0.015625	21.3333	−2.32727
CGT	39	0	4.39941	3.12618	−0	0.333333	0.015873	21	−2.33333
GNG	40	0	4.39941	3.12618	−0	0.333333	0.015873	21	−2.33333
TG	41	0	4.4371	3.13979	−0	0.333333	0.015625	21.3333	−2.32727
GT	42	0	2.34734	2.19478	−0	0.333333	0.046875	7.11111	−3.45946
T	43	0	1.85164	1.89481	−0	0.333333	0.0666667	5	−5
TC	45	0	2.74347	2.40997	−0	0.333333	0.0364583	9.14286	−2.97674
A	46	0	1.51136	1.66805	−0	0.333333	0.0871795	3.82353	−9.28571
AAG	48	0	5.4633	3.47682	−0	0.333333	0.010582	31.5	−2.21053
AG	49	0	5.82386	4.31461	−0	0.666667	0.0364583	18.2857	−2.39252
CAA	50	0	5.4633	3.47682	−0	0.333333	0.010582	31.5	−2.21053
AAG	51	0	5.4633	3.47682	−0	0.333333	0.010582	31.5	−2.21053
A	52	0	3.55799	3.17876	−0	0.666667	0.0871795	7.64706	−3.29114
T	53	0	1.85164	1.89481	−0	0.333333	0.0666667	5	−5
GG	55	0	4.4371	3.13979	−0	0.333333	0.015625	21.3333	−2.32727
T	56	0	1.85164	1.89481	−0	0.333333	0.0666667	5	−5
GGC	58	0	5.4633	3.47682	−0	0.333333	0.010582	31.5	−2.21053
GCA	59	0	5.4633	3.47682	−0	0.333333	0.010582	31.5	−2.21053
CCGG	60	0	7.76792	4.06346	−0	0.333333	0.00537634	62	−2.10169

## Data Availability

Data are contained within the research article.

## Supplementary Materials

The following supporting information can be downloaded at https://www.mdpi.com/article/10.3390/cells13191601/s1, Figure S1: The heatmap of the delta Ct (ΔCt) values for the investigated genes in each bull throughout the year. The color gradient in the heatmap (from light blue to dark blue) represents different ΔCt values for 10 genes, with darker colors indicating higher ΔCt values and lighter colors indicating lower ΔCt values. Old bull with 8x/9x GCC repeats and heterozygosity showed lowest delta Ct values particularly in the summer season for all genes. The zigzag pattern overlaid on certain cells for *NQO1* and *NRF1* represents genes that were not detected in the qPCR analysis.
