# Supplementary figures and images for: Genetic Variations in the NRF2 Microsatellite Contribute to the Regulation of Bovine Sperm-Borne Antioxidant Capacity
Source: Cells. 2024 Sep 24;13(19):1601. doi: 10.3390/cells13191601 (PMC11482559; doi:10.3390/cells13191601)

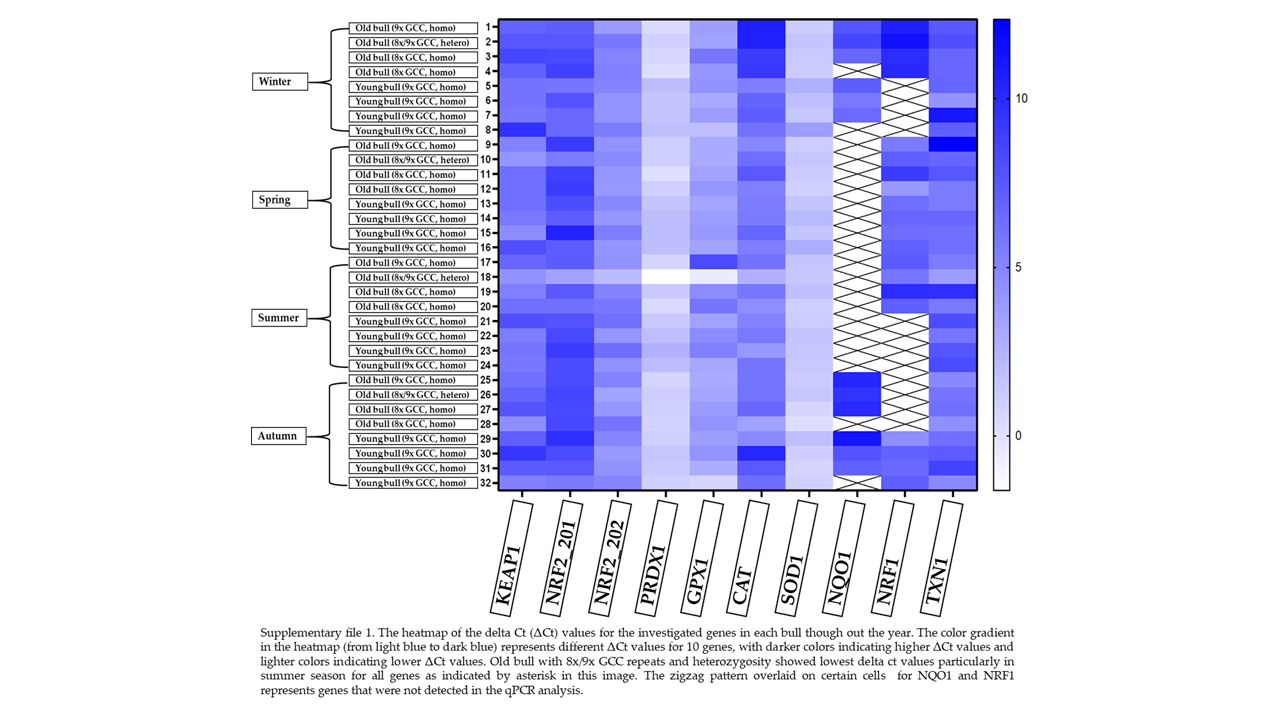

Supplement: Supplementary file 1 [file cells-13-01601-s001.zip › cells-3161675-supplementary.tif]
